# Exercise‐induced amplification of mitogen‐stimulated oxidative burst in whole blood is strongly influenced by neutrophil counts during and following exercise

**DOI:** 10.14814/phy2.15010

**Published:** 2021-09-08

**Authors:** A. Lester, G. L. Vickers, L. Macro, A. Gudgeon, A. Bonham‐Carter, J. P. Campbell, J. E. Turner

**Affiliations:** ^1^ Department for Health University of Bath Bath UK

**Keywords:** exercise, leukocytes, point‐of‐care systems, reactive oxygen species, respiratory burst

## Abstract

This study characterized the effect of moderate‐ or vigorous‐intensity exercise on leukocyte counts, using fingertip sampling, and mitogen‐stimulated oxidative burst, measured in whole blood with a point‐of‐care test. In a randomized crossover design, 13 healthy adults (mean ± SD age: 22 ± 2 years; seven male, six female) cycled for 30‐min, once at 52 ± 5% $\dot{V}$O_2peak_ and on another occasion at 74 ± 9% $\dot{V}$O_2peak_. Blood was sampled at baseline, immediately post‐exercise, and 15‐ and 60‐min post‐exercise. The leukocyte differential and mitogen‐stimulated Reactive Oxygen Species (ROS) production were assessed. Lymphocytes increased immediately post‐exercise and decreased below pre‐exercise levels 15‐ and 60‐min later. Lymphocyte mobilization immediately post‐exercise was 59 ± 36% greater with vigorous‐ compared to moderate‐intensity exercise (*p* < 0.01). Neutrophils increased immediately after exercise (38 ± 19%, *p* < 0.01) remaining elevated 60‐min later (50 ± 34%, *p* < 0.01; averaged across intensities) and did not differ between intensities (*p* = 0.259). Mitogen‐stimulated ROS production was amplified immediately (+32 ± 37%, *p* < 0.01) and 60‐min post‐exercise (+56 ± 57%, *p* < 0.01; averaged across intensities) compared to rest and did not differ with intensity (*p* = 0.739). Exercise‐induced amplification of ROS production was abolished when correcting for neutrophil, monocyte and platelet counts and correlated most strongly with neutrophil mobilization immediately (*r* = 0.709, *p* < 0.01) and 60‐min after vigorous exercise (*r* = 0.687, *p* < 0.01). Leukocyte kinetics can be assessed using fingertip blood sampling in exercise settings. Exercise‐induced amplification of oxidative burst is detectable with a point‐of‐care test, but results are strongly influenced by neutrophil counts, which may not be routinely quantified.

## 1 INTRODUCTION

It has consistently been demonstrated that acute bouts of exercise almost immediately increase the number of circulating leukocytes in peripheral blood (Gabriel et al., 1992; Nieman et al., 1989; Rooney et al., 2018), due to cells mobilizing from the endothelium, spleen and bone marrow which is thought to be an important part of immune surveillance (Dhabhar, 2014; Dhabhar et al., 2012). In the hours after exercise, although some cells such as neutrophils increase further, lymphocytes fall to approximately half of normal levels, returning to pre‐exercise values within 24 h (Gabriel et al., 1992; Nieman et al., 1989). It was originally thought that this decrease in circulating lymphocytes was a sign of immune suppression (Nieman et al., 1990; Nieman & Wentz., 2019). However, animal models have since shown that following exercise, highly functional sub‐types of lymphocytes extravasate to target organs, such as the lungs, where they are more likely to encounter pathogens (Krüger et al., 2008). Thus, the numerical count of leukocytes and their phenotypic composition in blood is drastically different when comparing measurements made before, during and after exercise.

Fluctuations in leukocytes and their subsets in blood strongly influence measurements of cell function after exercise (Campbell & Turner, 2018; Lancaster et al., 2005; Nieman et al., 1989). Among lymphocytes, although many studies have concluded that acute exercise bouts transiently impair T cell, B cell and NK cell function (Shaw et al., 2018; Siedlik et al., 2016), alterations in the proportions of specific cell subpopulations have often not been robustly accounted for in analyses (Campbell & Turner, 2018, 2019). Thus, there is limited evidence for exercise impairing the function of lymphocytes on a *per cell* basis (Simpson et al., 2020). However, conclusions over the impact that acute exercise bouts have on the function of other leukocyte subtypes are difficult to make due to variation in study design, assessment of different leukocyte functions, and varied assay conditions or measurement approaches (Beiter et al., 2014; Pyne, 1994; Suzuki et al., 1996).

Measuring the function of leukocytes in whole blood – rather than isolating specific cell types – has benefits, in part, because samples reflect possible contributing effects of soluble mediators and other cells or blood components which fluctuate during and following exercise. Subsequently, the need to consider blood sample composition, and in particular cell counts, could influence the precision of point‐of‐care tests that have been developed to assess aspects of immune function, especially if used in an exercise setting (McLaren et al., 2003; Shelton‐Rayner et al., 2010). For example, assays that measure mitogen‐stimulated oxidative burst in whole blood, may need to quantify and account for the predominant sources of reactive oxygen species (ROS) production in samples, including neutrophils, monocytes and platelets (Ghasemzadeh & Hosseini, 2017; Mantovani et al., 2011; Ponath & Kaina, 2017).

The aim of this study was to examine the effect of steady state cycling for 30 min at either moderate‐ or vigorous‐intensity on PMA‐stimulated ROS production in whole blood using fingertip sampling and a commercially available point‐of‐care assay. It was hypothesized that leukocyte counts and function would increase from baseline to immediately post‐exercise, and that greater responses would occur when exercise was of vigorous‐intensity (Robson et al., 1999). It was also hypothesized that exercise would amplify PMA‐stimulated ROS production, reflecting most predominantly, increases in the numerical count of neutrophils in blood (Suzuki et al., 1996).

## 2 METHODS

### 2.1 Participants

Thirteen participants (seven males, six females; mean ± SD age: 22 ± 2 years; BMI: 24.7 ± 3.0 kg m^−2^; $\dot{V}$O_2peak_: 44.8 ± 5.2 ml kg^−1^ min^−1^; body fat: 19.04 ± 7.94%) took part (Table S1). All participants were non‐smokers and self‐reported to be free from chronic disease, including cancer, cardiovascular disease, diabetes, auto‐immune and other inflammatory conditions. In the two weeks prior to participation, participants self‐reported that they had not developed an infection and were not taking any form of medication. Participants were asked to refrain from exercise, caffeine and alcohol for 24 h prior to the experimental trials.

### 2.2 Pre‐experimental procedures

Height was assessed using a stadiometer (Harpenden, Holtain Limited) and body mass was assessed using mechanical scales (Weylux). Body fat percentage was estimated using a four‐site skinfold assessment (bicep, tricep, subscapular and iliac crest) using skinfold callipers (Harpenden), following the methods of Durnin and Womersley (1974). Peak oxygen uptake ($\dot{V}$O_2peak_) was determined during exercise to volitional exhaustion on a cycle ergometer (Monark Peak 894E; Frayn, 1983). Following a 5‐min cycling warm‐up at 60 Watts, the load on the ergometer was increased by 30 Watts every 3‐min for the first 12‐min of the test, and every 2‐min thereafter, until exhaustion, maintaining 60 revolutions per minute (RPM) throughout. During the final minute of the first four stages and the final minute of the test, expired air samples were collected using Douglas bags (Hans Rudolph), heart rate was monitored (POLAR), and ratings of perceived exertion (RPE) were recorded using the Borg scale. Expired air samples were analysed for O_2_ and CO_2_ (Servomex). The final expired air sample was considered to be $\dot{V}$O_2peak_. Strong verbal encouragement was provided throughout the test. Oxygen uptake ($\dot{V}$O_2_) and work rate data were plotted to calculate the relative work rate to elicit 50% and 70% $\dot{V}$O_2peak_ for the experimental trials.

### 2.3 Experimental trials

At least 2 days after pre‐experimental procedures, participants completed their first experimental visit, arriving at the lab following an overnight fast between 08:00 and 10:00. Visits were undertaken in a randomized, counterbalanced order, with a 7‐day period between trials. Body mass was assessed upon arrival, and participants rested in a seated position for 15‐min. A resting fingertip blood sample was taken using a safety‐lancet (Sarstedt). Fingers from the same hand were sampled once per trial, and the order of blood sampling from the index, middle, and ring fingers (and hand) was randomized. After a 5‐min gentle warm up on the cycle ergometer at 60 Watts, work rate was adjusted to the predetermined 50% or 70% $\dot{V}$O_2peak_, and exercise continued at this intensity for 30‐min. Participants were instructed to maintain a cadence of 60 RPM throughout. At 5‐, 14‐, and 29‐min, expired air samples were collected for 1‐min and heart rate and RPE were monitored. Data were averaged across the three measurement points. Each exercise trial was conducted under similar environmental conditions (21°C), with a mechanical fan placed one meter away from the cycle ergometer and switched on at the lowest setting. Following exercise, participants remained seated. Immediately after exercise and 15‐, and 60‐min post‐exercise, a fingertip blood sample was collected.

### 2.4 Blood analysis

#### 2.4.1 Leukocyte count

Blood was collected in a 500 µL ethylene diaminetetraacetic acid (EDTA) tube (MiniCollect) and analysed immediately in duplicate for total leukocytes, lymphocytes, monocytes, neutrophils and platelets, using an automated hematology analyzer (Sysmex KX‐21N).

#### 2.4.2 Leukocyte production of reactive oxygen species

Leukocyte reactive oxygen species (ROS) production was assessed using a commercially available point‐of‐care assay (Leukocyte Coping Capacity, Oxford Medistress Ltd). Assay reagents were prepared 10 min prior to each blood sample by removing a lyophilized vial containing 10^−5^ M PMA, 10^−4^ M luminol and 0.1 U Heparin from a −20°C freezer and reconstituting with PBS according to manufacturer instructions. After mixing and centrifuging the vial for 30 s at 2000 *g*, 100 μL of this solution was added to a polystyrene antireflective luminometer tube and placed into a dry heat block at 37°C for 10‐min. For each sample, after the skin was pierced with a lancet, 10 μL of blood was pipetted directly from the finger into the luminometer tube, gently agitated for 2 s and incubated in the heat block at 37°C for a further 10‐min. After incubation, the tube was placed into a portable luminometer (Clean‐Trace, Gem Scientific) to assess relative light units (RLU) of chemiluminescence and thus production of ROS. Data are presented as absolute RLU and separately, RLU values corrected for the counts of neutrophils, monocytes and platelets (or in combination) due to their capacity to produce ROS (Ghasemzadeh & Hosseini., 2017; Mantovani et al., 2011; Ponath & Kaina, 2017).

### 2.5 Statistical analyses

Counts of total leukocytes, lymphocytes, monocytes, neutrophils, and platelets were corrected for blood volume changes as part of data analyses relating to frequency in blood (Bosch et al., 2005). However, when cell function data was adjusted, changes in neutrophils, monocytes and platelets, data were not corrected for blood volume changes. Data were assessed for normality using the Shapiro–Wilk test. Non‐normally distributed data were log transformed prior to statistical analysis. Differences at baseline between experimental conditions were assessed using paired sample *t* tests. A three‐way repeated measures Analysis of Variance (ANOVA), with a between groups factor to assess the effect of sex, was used to analyze differences across time and exercise intensity on leukocyte counts and PMA‐stimulated ROS production. Post‐hoc pairwise comparisons were performed using Bonferroni Stepwise adjustment. Pairwise comparisons were assessed using Student's *t* tests. Effect sizes from ANOVA models were reported as partial eta squared (*η*_p_^2^) and were considered as small ≥0.01 to <0.6, medium = 0.6 to <0.14, and large ≥0.14. For pairwise comparisons, effect sizes were reported as Cohen's *d* and interpreted as small ≥0.2 to 0.5, medium 0.5 to <0.8 and large ≥0.8. Statistical significance was considered to be *p* < 0.05. All data are expressed as mean ± standard deviation (SD) and were analysed using Microsoft Excel (Version 16.30) and IBM SPSS Statistics for Mac (Version 25).

## 3 RESULTS

### 3.1 Physiological and psychological responses to exercise

All participants completed both experimental trials. Summary data for % $\dot{V}$O_2peak_, work rate, heart rate, $\dot{V}$O_2_, respiratory exchange ratio (RER) and RPE are shown in Table 1. Participants exercised at 52 ± 5% $\dot{V}$O_2peak_ during the 50% $\dot{V}$O_2peak_ trial, and at 74 ± 9% $\dot{V}$O_2peak_ during the 70% $\dot{V}$O_2peak_ trial (*t*_(12)_ = −13.117, *p* < 0.01, *d* = 3.06). As expected, work rate, heart rate, $\dot{V}$O_2_, RER, and RPE were significantly higher in the 70% $\dot{V}$O_2peak_ trial (all *p* < 0.05). Males and females exercised at a similar % $\dot{V}$O_2peak_ in each trial, and all other parameters were significantly higher in the 70% compared to 50% $\dot{V}$O_2peak_ trial when analysed separately for each sex, except for RER among males (Table S2). On this basis, immunological data is presented for all participants combined (*n* = 13) but sex‐specific statistical analysis is briefly summarized where appropriate and shown in Table S3.

**TABLE 1:** Exercise physiology data across the two exercise intensities

	50% $\dot{V}$O_2peak_	70% $\dot{V}$O_2peak_
% VO_2peak_	52 ± 5	74 ± 9*
Work rate (Watts)	117 ± 34	181 ± 59 *
Heart rate (bpm)	124 ± 14	157 ± 13 *
$\dot{V}$O_2_ (L/min)	1.80 ± 0.45	2.61 ± 0.78 *
RER	0.91 ± 0.07	0.97 ± 0.05 *
RPE	10 ± 1	13 ± 2 *

Note

Values are mean ± SD.

Abbreviations: RER, respiratory exchange ratio; RPE, rating of perceived exertion.

* *p* < 0.05, denotes a significant difference between the two exercise intensities.

### 3.2 Total leukocyte, lymphocyte, monocyte, neutrophil, and platelet count in response to exercise

**FIGURE 1:**
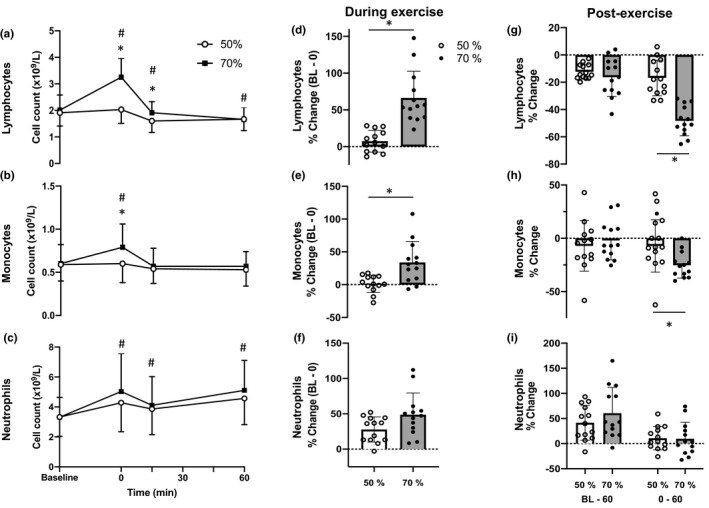
Panels A‐C show absolute counts of cells before and after exercise. Panels D‐F show percentage change in cell counts during exercise, calculated between baseline and immediately post exercise. Panel G‐I show percentage change in cell counts after exercise, calculated between baseline and 60 min post, or between immediately post and 60 min post. Values are mean ± SD. BL refers to baseline which was assessed pre‐exercise. 0, 15, 60 refer to minutes post exercise. *p < 0.05 indicates a significant difference between exercise intensities, and ^#^ *p* < 0.05 indicates a significant difference compared to baseline across both intensities combined, determined by post hoc Bonferroni Stepwise analyses

Total leukocyte, lymphocyte, monocyte, neutrophil, and platelet counts changed across time and there was a significant time × intensity interaction effect for all cells except neutrophils and platelets (Table 2; Figure 1a–c). Immediately after exercise at 50% $\dot{V}$O_2peak_, lymphocyte count increased by 7.4 ± 14.8%, whereas at 70% $\dot{V}$O_2peak_ lymphocytes increased by 66.4 ± 36.2% (*t*_(12)_ = −5.546, *p* < 0.01, *d* = 2.13; Figure 1d). Lymphocyte egress from baseline to 60‐min post‐exercise was not significantly different between the 70% $\dot{V}$O_2peak_ (−16.3 ± 14.1%), and the 50% $\dot{V}$O_2peak_ trials (−12.3 ± 5.5%; *t*_(12)_ = 0.961, *p* = 0.356, *d* = 0.37; Figure 1g). However, egress was significantly different between the intensities when assessed from immediately post‐exercise to 60‐min post‐exercise, with lymphocytes decreasing by −48.3 ± 10.9% in the 70% $\dot{V}$O_2peak_ trial and decreasing by −16.8 ± 13.0% in the 50% $\dot{V}$O_2peak_ trial (*t*_(12)_ = −9.082, *p* < 0.01, *d* = 2.89; Figure 1g).

**TABLE 2:** Changes in total leukocyte, lymphocyte, monocyte, and neutrophil count in response to moderate and vigorous intensity exercise

	Baseline	0	15	60	Main effect of time	Interaction effect of intensity × time
Leukocytes						
50%	5.8 ± 1.4	6.9 ± 2.2^#^	6.0 ± 1.8	6.8 ± 1.8^#^	*F*_(3, 9)_ = 27.453 *p* < 0.01 *η*_p_^2^ = 0.714	*F*_(3, 9)_ = 7.932, *p* < 0.01, *η*_p_^2^ = 0.340
70%	5.9 ± 1.5	9.1 ± 2.9^#^ ^,^ *	6.6 ± 2.1	7.3 ± 2.1^#^		
Lymphocytes						
50%	1.9 ± 0.5	2.0 ± 0.5	1.6 ± 0.4	1.7 ± 0.4^#^	*F*_(3, 9)_ = 73.421, *p* < 0.01, *η*_p_^2^ = 0.870	*F*_(3, 9)_ = 46.776, *p* < 0.01, *η*_p_^2^ = 0.810
70%	2.0 ± 0.6	3.2 ± 0.7^#^ ^,^ *	1.9 ± 0.4*	1.7 ± 0.5^#^		
Monocytes						
50%	0.6 ± 0.2	0.6 ± 0.2	0.5 ± 0.2	0.5 ± 0.2	*F*_(3, 9)_ = 12.932, *p* < 0.01, *η*_p_^2^ = 0.540	*F*_(3, 9)_ = 5.447, *p* < 0.01, *η*_p_^2^ = 0.331
70%	0.6 ± 0.2	0.8 ± 0.3^#^ ^,^ *	0.6 ± 0.2	0.6 ± 0.2		
Neutrophils						
50%	3.3 ± 1.3	4.3 ± 1.9	3.9 ± 1.7^#^	4.6 ± 1.7^#^	*F*_(3, 9)_ = 23.347, *p* < 0.01, *η*_p_^2^ = 0.680	*F*_(3, 9)_ = 1.404, *p* = 0.259, *η*_p_^2^ = 0.113
70%	3.3 ± 1.3	5.0 ± 2.5^#^	4.1 ± 1.9	5.1 ± 2.0^#^		
Platelets						
50%	1.9 ± 0.4	2.0 ± 0.6^#^	1.9 ± 0.5	1.8 ± 0.5	*F*_(3, 9)_ = 22.359, *p* < 0.01, *η*_p_^2^ = 0.651	*F*_(3, 9)_ = 2.497, *p* = 0.111, *η*_p_^2^ = 0.172
70%	1.9 ± 0.5	2.4 ± 0.7^#^	2.1 ± 0.6^#^	1.9 ± 0.5		

Note

Values are mean ± SD. 0, 15, and 60 refer to minutes post exercise.

* *p* < 0.05 indicates a significant difference between exercise intensities.

# *p* < 0.05 indicates a significant difference compared to baseline, where a main effect of time was found for each intensity, determined by post hoc Bonferroni Stepwise analyses. Of monocytes, <10% will be basophils and eosinophils. Leukocytes, lymphocytes, monocytes, and neutrophils are presented as ×10^9^/L and platelets are presented as ×10^7^/L.

Immediately after exercise at 50% $\dot{V}$O_2peak_, monocyte count increased by 1.4 ± 13.3%, whereas at 70% $\dot{V}$O_2peak_ monocytes increased by 33.9 ± 32.0% (*t*_(12)_ = −3.305, *p* < 0.01, *d* = 1.32; Figure 1e). Monocyte egress from baseline to 60‐min post‐exercise was not significantly different between the 70% $\dot{V}$O_2peak_ (−2.0 ± 18.3%), and the 50% $\dot{V}$O_2peak_ trial (−7.1 ± 23.7%; *t*_(12)_ = −0.598, *p* = 0.561, *d* = 0.24; Figure 1h). However, egress was significantly different between the intensities when assessed from immediately post‐exercise to 60‐min post‐exercise. Exercise at 70% $\dot{V}$O_2peak_ decreased monocytes by −25.3 ± 12.1% whereas the 50% $\dot{V}$O_2peak_ trial caused a decrease of −7.1 ± 24.6% (*t*_(12)_ = 2.543, *p* < 0.05, *d* = 0.94; Figure 1h).

Immediately after exercise at 50% $\dot{V}$O_2peak_, neutrophil count increased by 27.8 ± 17.8%, whereas at 70% $\dot{V}$O_2peak_ neutrophils increased by 48.8 ± 30.8% (*t*_(12)_ = −2.147, *p* = 0.053, *d* = 0.83; Figure 1f). The increase in neutrophil count from baseline to 60‐min post‐exercise was not significantly different between the 70% $\dot{V}$O_2peak_ trial (60.6 ± 51.4%), and the 50% $\dot{V}$O_2peak_ trial (41.5 ± 34.6%; *t*_(12)_ = −1.264, *p* = 0.230, *d* = 0.44; Figure 1i). Likewise, increases in neutrophil count were not significantly different between the intensities when assessed from immediately post‐exercise to 60‐min post‐exercise (70% $\dot{V}$O_2peak_: 9.4 ± 33.2% and 50% $\dot{V}$O_2peak_: 10.8 ± 23.3%, *t*_(12)_ = 0.139, *p* = 0.892, *d* = 0.05; Figure 1i).

Immediately after exercise at 50% $\dot{V}$O_2peak_, platelet count increased by 11.9 ± 15.1%, whereas at 70% $\dot{V}$O_2peak_, platelets increased by 18.5 ± 21.5% (*t*_(12)_ = −1.572, *p* = 0.116, *d* = 0.61). During the 50% $\dot{V}$O_2peak_ trial, platelet count decreased by 3.9 ± 16.6% from baseline to 60 min post exercise, whereas during the 70% $\dot{V}$O_2peak_ trial, platelet count increased marginally by 5.6 ± 7.8% (*t*_(12)_ = −0.175, *p* = 0.861, *d* = 0.19). Decreases in platelet count were not significantly different between intensities when assessed from immediately post‐exercise to 60‐min post exercise (50% $\dot{V}$O_2peak_ 12.1 ± 22.7% and 70% $\dot{V}$O_2peak_ 11.7 ± 15.3%, *t*_(12)_ = −1.433, *p* = 0.152, *d* = 0.30; Table 2).

There was no main effect of sex on leukocyte (*F*_(1, 11)_ = 0.11, *p* = 0.746, *η*_p_^2^ = 0.010), lymphocyte (*F*_(1, 11)_ = 0.627, *p* = 0.445, *η*_p_^2^ = 0.054), monocyte (*F*_(1, 11)_ = 2.585, *p* = 0.136, *η*_p_^2^ = 0.190), neutrophil count (*F*_(1, 11)_ = 0.182, *p* = 0.678, *η*_p_^2^ = 0.016) or platelet count (*F*_(1, 11)_ = 1.275, *p* = 0.283, *η*_p_^2^ = 0.104). The only time × intensity interaction effect that was statistically significant when analyzed separately for each sex was for lymphocytes, and there were no statistically significant time × intensity × sex interaction effects for any cells (Table S3).

### 3.3 PMA‐stimulated ROS production expressed as absolute values

**FIGURE 2:**
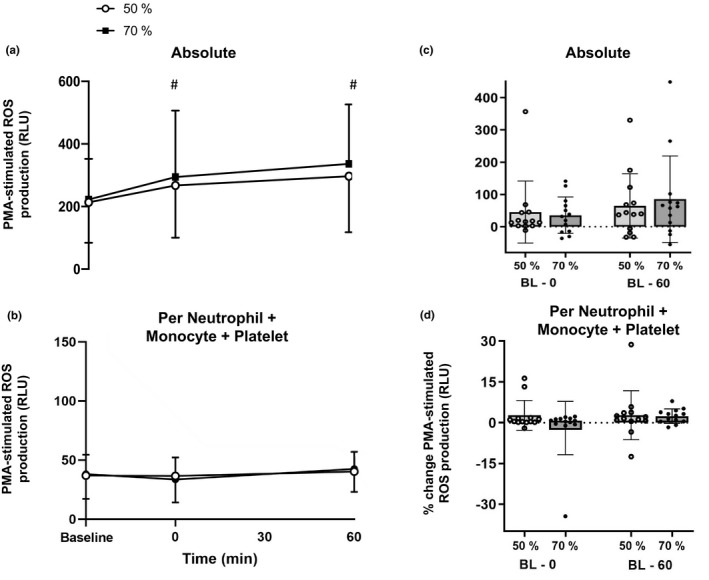
Panel A shows PMA‐stimulated ROS production during and after exercise with data expressed as absolute values. Panel B shows PMA‐stimulated ROS production during and after exercise with data expressed relative to a composite score of neutrophil, monocyte and platelet counts. Panel C and D show data from Panel A and B expressed as percentage change, calculated between baseline and immediately post exercise, or baseline and 60 min post exercise. Values are mean ± SD. BL refers to baseline which was assessed pre‐exercise. 0, 30, 60 refer to minutes post exercise. ^#^ *p* < 0.05 indicates a significant difference compared to baseline across both intensities combined, determined by post hoc Bonferroni Stepwise analyses

There was a statistically significant main effect of time for PMA‐stimulated ROS production (*F*_(2, 10)_ = 6.069, *p* = 0.008, *η*_p_^2^ = 0.356; Figure 2a) which increased from baseline to immediately post‐exercise (*t*_(12)_ = −2.892, *p* = 0.008, *d* = 0.39) and from baseline to 60‐min post‐exercise (*t*_(12)_ = −3.762, *p* < 0.001, *d* = 0.63). There was no significant time × intensity interaction effect (*F*_(2, 10)_ = 0.307, *p* = 0.739, *η*_p_^2^ = 0.027). When PMA‐stimulated ROS production was expressed as percentage change, there were no differences between trials from baseline to immediately post‐exercise (50% $\dot{V}$O_2peak_ Δ + 45 ± 95% and 70% $\dot{V}$O_2peak_ Δ + 35 ± 100%) or between baseline and 60‐min post‐exercise (50% $\dot{V}$O_2peak_ Δ + 64 ± 100% and 70% $\dot{V}$O_2peak_ Δ+85 ± 134%; Figure 2c). There was no time × sex interaction effect (*F*_(1, 11)_ = 0.920, *p* = 0.409, *η*_p_^2^ = 0.077) or time × intensity × sex interaction effect (*F*_(1, 11)_ = 0.762, *p* = 0.470, *η*_p_^2^ = 0.065; data not shown).

### 3.4 PMA‐stimulated ROS production expressed relative to neutrophil, monocyte, and platelet counts

When PMA‐stimulated ROS production was expressed per neutrophil, the main effect of time became non‐significant (*F*_(1, 11)_ = 1.834, *p* = 0.183, *η*_p_^2^ = 0.143). However, when correcting for monocytes or platelets the main effect of time strengthened (monocytes: *F*_(1, 11)_ = 12.6 *p* = 0.002, *η*_p_^2^ = 0.534; platelets: *F*_(1, 11)_ = 7.221, *p* = 0.004 *η*_p_^2^ = 0.396). Given the influence that neutrophils, monocytes, and platelets had on PMA‐stimulated ROS production, this combined effect was corrected for using a composite score (i.e., combined counts of neutrophils + monocytes + platelets) and the main effect of time became non‐significant (*F*_(1, 11)_ = 2.303, *p* = 0.130, *η*_p_^2^ = 0.161; Figure 2b,d). Correcting for other combinations showed that neutrophil count had the strongest influence on PMA‐stimulated ROS production, as the main effects of time were lost (neutrophils + monocytes: *F*_(1, 11)_ = 1.449, *p* = 0.257, *η*_p_^2^ = 0.116; and neutrophils + platelets: *F*_(1, 11)_ = 1.820, *p* = 0.189, *η*_p_^2^ = 0.132). When correcting for the combination of monocytes + platelets, the main effect of time remained significant (*F*_(1, 11)_ = 8.543, *p* = 0.002, *η*_p_^2^ = 0.416). There were no time × intensity interaction effects for PMA‐stimulated ROS production corrected separately for neutrophils, monocytes, and platelets or their combinations (*F*_(1, 11)_ = 0.200 to 1.482, *p* = 0.252 to 0.658, *η*_p_^2^ = 0.018 to 0.119). Across all corrections, there were no time × sex interaction effects (*F*_(1, 11)_ = 0.595 to 3.891, *p* = 0.059 to 0.556, *η*_p_^2^ = 0.051 to 0.261), or time × intensity × sex interaction effects (*F*_(1, 11)_ = 0.461 to 1.318, *p* = 0.287 to 0.549, *η*_p_^2^ = 0.040 to 0.107; data not shown).

### 3.5 Relationship between PMA‐stimulated ROS production and neutrophil, monocyte and platelet counts

There was a positive correlation between percentage change in PMA‐stimulated ROS production and percentage change in neutrophil count from baseline to immediately post‐exercise (*r* = 0.709, *p* = 0.007) and from baseline to 60‐min post exercise (*r* = 0.687, *p* = 0.01) in the 70% $\dot{V}$O_2peak_ trial (Figure S1A,D). The relationship was not statistically significant in the 50% $\dot{V}$O_2peak_ trial from baseline to immediately post‐exercise (*r* = 0.302, *p* = 0.316) but was significant between baseline and 60‐min post exercise (*r* = 0.621, *p* = 0.024; Figure S1D).

There was a positive correlation between percentage change in PMA‐stimulated ROS production and percentage change in monocyte count, from baseline to immediately post‐exercise (*r* = 0.590, *p* = 0.034) in the 70% $\dot{V}$O_2peak_ trial (Figure S1B). The relationship was not statistically significant in the 50% $\dot{V}$O_2peak_ trial from baseline to immediately post‐exercise (*r* = 0.590, *p* = 0.564). There were no significant correlations between percentage change in monocyte count and PMA‐stimulated ROS production from baseline to 60‐min post‐exercise (*r* < 0.376, *p* > 0.206; Figure S1E).

There was a positive correlation between percentage change in PMA‐stimulated ROS production and percentage change in platelet count, from baseline to immediately post‐exercise in the 70% $\dot{V}$O_2peak_ trial (*r* = 0.609, *p* = 0.027) but not the 50% $\dot{V}$O_2peak_ trial (*r* = 0.357, *p* = 0.231; Figure S1C). There were no significant correlations with change in platelet count for either intensities between baseline and 60‐min post exercise (*r* < 0.352, *p* > 0.239; Figure S1F).

**FIGURE 3:**
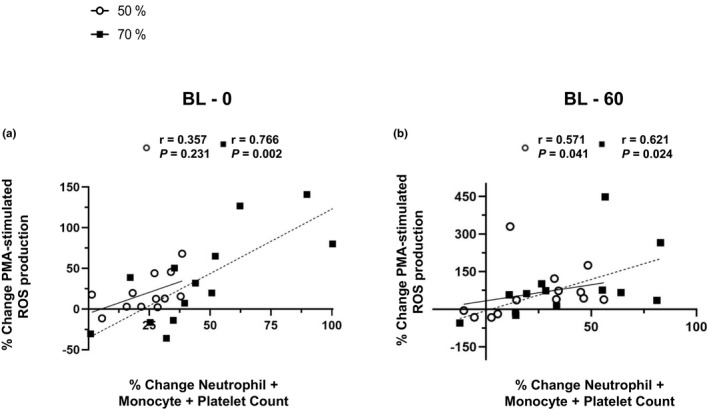
(a) Correlation between the percentage change in a composite score of neutrophils + monocytes + platelets and the percentage change in PMA‐stimulated ROS production from baseline to 0 (immediately post exercise). (b) Data are shown from baseline to 60 min post exercise

Figure 3a shows that there was a positive correlation between percentage change in PMA‐stimulated ROS production and percentage change in the combined counts of neutrophils + monocytes + platelets, from baseline to immediately post‐exercise in the 70% $\dot{V}$O_2peak_ trial (*r* = 0.766, *p* = 0.002) but there was no relationship in the 50% $\dot{V}$O_2peak_ trial (*r* = 0.357, *p* = 0.231). There was positive correlation between percentage change in PMA‐stimulated ROS production and percentage change in the combined counts of neutrophils + monocytes + platelets, from baseline to 60‐min post‐exercise in both trials (*r* > 0.570, *p* < 0.041; Figure 3b).

## 4 DISCUSSION

This study characterized the effect of exercise on leukocyte counts using fingertip sampling, and mitogen‐stimulated oxidative burst, measured in whole blood with a point‐of‐care test. Exercise elicited the expected and well‐characterized changes to the counts of leukocytes and platelets. PMA‐stimulated ROS production in whole blood was increased immediately after exercise, remaining high for 1 h. When the increase in PMA‐stimulated ROS production was expressed relative to the increase in neutrophils, monocytes and platelets – key ROS producers in whole blood – the amplification effect of exercise was abolished. The increase in PMA‐simulated ROS production in whole blood was most strongly correlated with the increase in neutrophils, indicating that exercise bouts are capable of amplifying oxidative burst assessed in whole blood, but this is largely brought about by exercise‐induced increases to cell counts.

The influence that acute exercise bouts have on leukocyte counts is well‐investigated. To establish whether these highly reproducible effects were measurable with fingertip blood sampling, this study examined both moderate‐and vigorous‐intensity exercise, and the expected biphasic change to the counts of leukocytes and their subtypes was shown across both intensities. Compared to moderate‐intensity exercise, vigorous‐intensity exercise elicited the most pronounced effects, but only among lymphocytes, aligning with previous studies (Neves et al., 2015). In the present study, there was not a differential effect of exercise intensity on neutrophils, however this phenomenon has been reported previously. For example, Robson et al. (1999) showed that neutrophil count was greater following cycling exercise to exhaustion at 80% $\dot{V}$O_2max_ compared to cycling exercise for 3 h at 55% $\dot{V}$O_2max_. Similar findings were shown by Thammawong et al. (2017) by comparing exercise bouts that were substantially different in terms of exercise intensity: cycling at 90% $\dot{V}$O_2max_ compared to 30% $\dot{V}$O_2max_. It is possible that a differential effect of moderate‐ (52 ± 5% $\dot{V}$O_2peak_) versus vigorous‐intensity (74 ± 9% $\dot{V}$O_2peak_) exercise on neutrophil counts was not shown in the present study due to the smaller difference in exercise intensity compared to previous investigations.

This study showed that PMA‐stimulated ROS production in whole blood increased immediately after exercise and remained high for 1 h. However, when data were corrected for the change in sample composition considering key ROS producers in blood (i.e. neutrophils, monocytes and platelets) the amplifying effects of exercise were abolished. Similar findings have been reported previously. For example, Suzuki et al. (1996) reported an increase in zymosan‐stimulated ROS production among isolated neutrophils using luminol‐amplified chemiluminescence immediately after incremental treadmill running to exhaustion. This exercise‐induced amplification of ROS production was significantly correlated (*r* = 0.669) with neutrophil count. However, other studies have reported different results, including a decrease in PMA‐stimulated oxidative burst in whole blood assessed using flow cytometry and the probe hydroethidine following cycling exercise at both 80% $\dot{V}$O_2max_ (37 ± 19 min) and at 55% $\dot{V}$O_2max_ for 3 h (Robson et al., 1999). Conclusions over the impact that acute exercise bouts have on measurements of leukocyte function are difficult to make, primarily due to varied assay conditions and measurement approaches (Beiter et al., 2014; Pyne, 1994; Suzuki et al., 1996).

The results of this study show that changes to leukocyte function can be assessed in the context of exercise using fingertip blood sampling and a commercially available point‐of‐care test. The functional assay used in the present study has the advantage of working with a stimulant that is commonly used in laboratory‐based assays (i.e. PMA which stimulates ROS production via NADPH oxidase activation; Bedouhène et al., 2017; Karlsson et al., 2000), but in an easy‐to‐use, readily available format that can be employed in non‐laboratory settings. Using whole blood has other advantages, such as avoiding non‐specific cell activation which can occur with some blood processing and cell separation techniques (Himmelfarb et al., 1992). Further, as the test used in the present study is compatible with small blood volumes obtained via fingertip samples, which has previously been shown to be reflective of venous sampling (Canetti et al., 2016), this kit provides greater accessibility to functional tests. However, it should be considered that this method is yet to be validated against other measurements of neutrophil function, including, for example, a test that is considered to be a “gold standard measure” (i.e. counting colony forming units; van Grinsven et al., 2018). It should also be emphasized that even with simple measurements of leukocyte function that are compatible with fingertip sampling and point‐of‐care methodology, it is essential to consider cell counts, to avoid over‐interpreting “immune‐boosting” effects of exercise (Campbell & Turner, 2018). Thus, for other studies using point‐of‐care leukocyte function assays, a dual platform approach might be needed, whereby leukocyte function is assessed in parallel with the leukocyte differential, which can also be assessed with portable devices (Karawajczyk et al., 2017).

In the present study, males and females took part, but sex differences in leukocyte kinetics and PMA‐stimulated ROS production were not shown. A limitation is that the menstrual cycle phase was not controlled for which has previously been shown to influence the immune response to exercise (Davis et al., 2000; Timmons et al., 2005). As with other studies (Hack et al., 1992; Robson et al., 1999; Suzuki et al., 1996), the present work did not account for proportional shifts in neutrophil sub‐types. For example, the neutrophils which are rapidly responsive to exercise mobilize from the endothelium and are mature, whereas at other post‐exercise time‐points, immature cells may mobilize from bone marrow (Smith et al., 1996). Given that immature neutrophils have an impaired capacity to produce ROS (Pillay et al., 2010), future studies should quantify the proportions of mature and immature neutrophils in samples. Another limitation of the present work is that we did not examine whether other characteristics of our samples, such as the post‐exercise inflammatory milieu, could have influenced cell function. Indeed, it has previously been shown that circulating pro‐inflammatory cytokines, such as tumor necrosis factor‐alpha (TNF‐*α*), primes neutrophil ROS production (Elbim et al., 1994). A final consideration is that the point‐of‐care assay used in this investigation (Leukocyte Coping Capacity, Oxford Medistress Ltd, Oxford, UK) is limited to the assessment of ROS production in whole blood stimulated with PMA and does not include an unstimulated control. Thus, analyses cannot quantify the effects of exercise on basal ROS production and are therefore limited to establishing whether there is a further exercise‐induced amplification effect following stimulation.

In summary, the present study showed that 30 min of cycling exercise elicited the expected biphasic changes to the counts of leukocytes and their subtypes, as well as the expected changes to neutrophil function measured using fingertip blood sampling and a commercially available point‐of‐care test. Both intensities of exercise amplified PMA‐stimulated ROS production, primarily by increasing neutrophil counts.

## CONFLICT OF INTEREST

The authors declare no competing interests.

## AUTHOR CONTRIBUTIONS

**Alice Lester:** Conceptualization, Methodology, Formal analysis, Investigation, Visualization Preparation, Writing ‐ Original Draft, Writing ‐ Review & Editing. **Gabrielle Vickers:** Conceptualization, Methodology, Investigation, Writing ‐ Review & Editing. **Laura Macro:** Conceptualization, Methodology, Investigation, Writing ‐ Review & Editing. **Alex Gudgeon:** Conceptualization, Methodology, Investigation. **Alice Bonham‐Carter:** Conceptualization, Methodology, Investigation. **John Campbell:** Resources, Funding acquisition, Writing ‐ Review & Editing. **James Turner:** Conceptualization, Methodology, Formal analysis, Resources, Visualization Preparation, Project administration, Funding acquisition, Supervision, Writing ‐ Original Draft, Writing ‐ Review & Editing.

## ETHICAL APPROVAL

The research complied with all relevant federal guidelines and institutional policies. The study was approved by the Research Ethics Approval Committee for Health (REACH) at the University of Bath (reference: BSCFYP 19/20‐020) and participants provided written, informed consent.

## Supporting information



Fig S1

Table S1‐S3

## Data Availability

Data created during this research is openly available at the University of Bath Research Data Archive:https://researchdata.bath.ac.uk/id/eprint/1070
