# Supplementary material for: Exercise‐induced amplification of mitogen‐stimulated oxidative burst in whole blood is strongly influenced by neutrophil counts during and following exercise
Source: Physiol Rep. 2021 Sep 8;9(17):e15010. doi: 10.14814/phy2.15010 (PMC8425910; doi:10.14814/phy2.15010)
Supplement: Supplementary file 1 — Fig S1 [file PHY2-9-e15010-s002.pptx]

## Slide 1
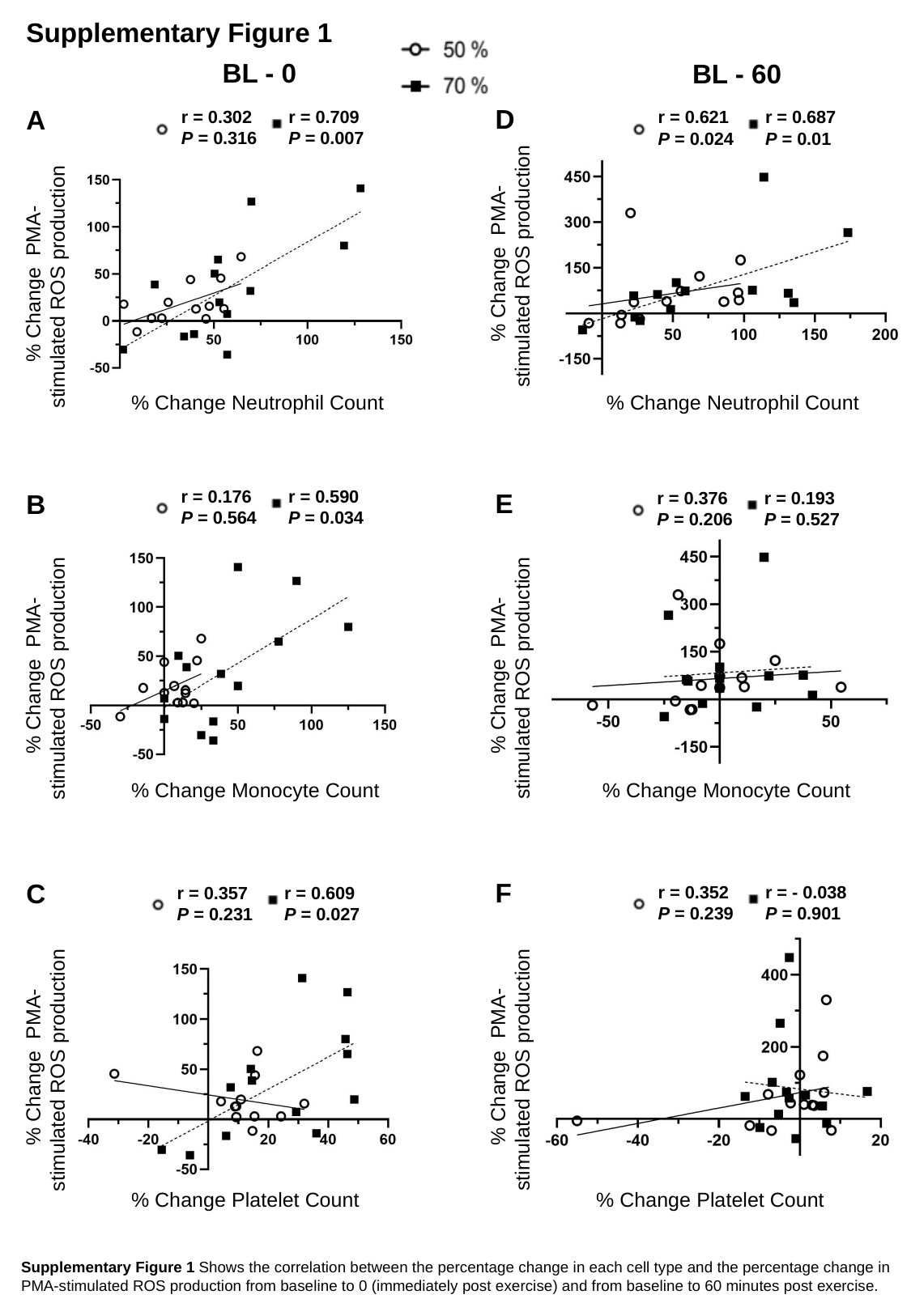

Supplementary Figure 1
BL - 0
BL - 60
D
A
r = 0.302
P = 0.316
r = 0.709
P = 0.007
r = 0.621
P = 0.024
r = 0.687
P = 0.01
 % Change PMA-stimulated ROS production
 % Change PMA-stimulated ROS production
% Change Neutrophil Count
% Change Neutrophil Count
r = 0.176
P = 0.564
r = 0.590
P = 0.034
E
r = 0.376
P = 0.206
B
r = 0.193
P = 0.527
 % Change PMA-stimulated ROS production
 % Change PMA-stimulated ROS production
% Change Monocyte Count
% Change Monocyte Count
F
C
r = 0.352
P = 0.239
r = - 0.038
P = 0.901
r = 0.357
P = 0.231
r = 0.609
P = 0.027
 % Change PMA-stimulated ROS production
 % Change PMA-stimulated ROS production
% Change Platelet Count
% Change Platelet Count
Supplementary Figure 1 Shows the correlation between the percentage change in each cell type and the percentage change in PMA-stimulated ROS production from baseline to 0 (immediately post exercise) and from baseline to 60 minutes post exercise.
