# Supplementary material for: Exercise‐induced amplification of mitogen‐stimulated oxidative burst in whole blood is strongly influenced by neutrophil counts during and following exercise
Source: Physiol Rep. 2021 Sep 8;9(17):e15010. doi: 10.14814/phy2.15010 (PMC8425910; doi:10.14814/phy2.15010)
Supplement: Supplementary file 2 — Table S1‐S3 [file PHY2-9-e15010-s001.docx]

Supplementary Tables

Supplementary Table 1. Participant characteristics

|  | All (N = 13) | Males (N = 7) | Females (N = 6) |
| --- | --- | --- | --- |
| Age (years) | 22 ± 2 | 22 ± 3 | 22 ± 1 |
| Height (cm) | 176.8 ± 12.2 | 185.6 ± 8.2 | 166.5 ± 6.2 * |
| Body mass (kg) | 77.9 ± 16.0 | 89.1 ± 12.6 | 64.9 ± 6.6 * |
| BMI (kg.m^-2^) | 24.7 ± 3.0 | 25.9 ± 3.7 | 23.3 ± 0.8 |
| V̇O_2peak_ (mL.kg^-1^.min^-1^) | 44.8 ± 5.2 | 48.6 ± 3.7 | 40.5 ± 2.9 * |
| Body fat (%) | 19.04 ± 7.94 | 12.46 ± 2.21 | 26.74 ± 3.66 * |

Values are mean ± standard deviation (SD). * (*P* < 0.05) denotes significantly different from males.

Supplementary Table 2. Exercise physiology data across the two exercise intensities

|  | 50 % | | 70 % | |
| --- | --- | --- | --- | --- |
|  | M | F | M | F |
| % VO_2peak_ | 50 ± 5 | 54 ± 5 | 73 ± 11 **^†^** | 76 ± 7 **^#^** |
| Work Rate (Watts) | 141 ± 26 | 88.31 ± 13 * | 223 ± 49 **^†^** | 132 ± 18 ***^#^** |
| Heart Rate (bpm) | 122 ± 10 | 129 ± 11* | 152 ± 15 **^†^** | 150 ± 8 **^#^** |
| V̇O_2_ (L/min) | 2.15 ± 0.28 | 1.40 ± 0.16 * | 3.15 ± 0.65 **^†^** | 1.97 ± 0.19 ***^#^** |
| RER | 0.90 ± 0.09 | 0.92 ± 0.05 | 0.96 ± 0.06 **^†^** | 0.99 ± 0.03 **^#^** |
| RPE | 10 ± 1 | 11 ± 0 * | 13 ± 2 | 13 ± 2 **^#^** |

Values are mean ± SD. M = males, F = females. **^†^** for males, and **^#^** for females (both *P* < 0.05), denotes a significant difference between 70 % and 50 % V̇O_2peak_. * (*P* < 0.05) denotes females were significantly different from males within the same exercise intensity trial.

| Supplementary Table 3. Changes in total leukocyte, lymphocyte, mixed cell, neutrophil, and platelet count in response to moderate and vigorous intensity exercise. | 50% V̇O_2peak_ | | | | 70% V̇O_2peak_ | | | | Main effect of time | Time x Intensity | Time x Intensity x Sex |
| --- | --- | --- | --- | --- | --- | --- | --- | --- | --- | --- | --- |
| Cell | Baseline | 0 | 15 | 60 | Baseline | 0 | 15 | 60 |  |  |  |
| ***Leukocytes*** |  |  |  |  |  |  |  |  |  |  | *F* = 0.392  *P* = 0.680  η_p_^2^ = 0.034 |
| Male | 6.3 ± 1.5 | 7.2 ± 2.9 | 6.3 ± 2.2 | 6.9 ± 2.4 | 6.3 ± 1.9 | 9.2 ± 3.3 | 6.5 ± 2.1 | 6.9 ± 2.0 | *F* = 11.188  *P* < 0.01  η_p_^2^ = 0.651 | *F* = 4.80  *P* < 0.05  η_p_^2^ = 0.445 |  |
| Female | 5.2 ± 1.1 | 6.7 ± 1.3 | 5.6 ± 1.1 | 6.6 ± 0.9 | 5.6 ± 0.9 | 8.9 ± 2.7 | 6.6 ± 2.4 | 7.8 ± 2.3 | *F* = 18.808  *P* < 0.01  η_p_^2^ = 0.790 | *F* = 3.024  *P* = 0.062  η_p_^2^ = 0.377 |  |
| ***Lymphocytes*** |  |  |  |  |  |  |  |  |  |  | *F* = 0.090  *P* = 0.932  η_p_^2^ = 0.008 |
| Male | 1.9 ± 0.6 | 1.9 ± 0.5 | 1.6 ± 0.5 | 1.6 ± 0.5 | 1.9 ± 0.6 | 3.0 ± 0.7 | 1.9 ± 0.5 | 1.6 ± 0.6 | *F* = 28.554  *P* < 0.01  η_p_^2^ = 0.826 | *F* = 37.720  *P =* < 0.01  η_p_^2^ = 0.863 |  |
| Female | 2.0 ± 0.5 | 2.2 ± 0.4 | 1.7 ± 0.3 | 1.7 ± 0.4 | 2.1 ± 0.6 | 3.5 ± 0.7 | 2.0 ± 0.3 | 1.8 ± 0.3 | *F* = 45.933  *P* < 0.01  η_p_^2^ = 0.902 | *F* = 15.515  *P* < 0.01  η_p_^2^ = 0.756 |  |
| ***Monocytes*** |  |  |  |  |  |  |  |  |  |  | *F* = 0.379  *P* = 0.724  η_p_^2^ = 0.033 |
| Male | 0.7 ± 0.2 | 0.7 ± 0.3 | 0.6 ± 0.2 | 0.6 ± 0.2 | 0.7 ± 0.3 | 0.8 ± 0.3 | 0.7 ± 0.2 | 0.6 ± 0.2 | *F* = 4.916  *P* < 0.05  η_p_^2^ = 0.450 | *F* = 3.579  *P* = 0.053  η_p_^2^ = 0.374 |  |
| Female | 0.5 ± 0.1 | 0.5 ± 0.1 | 0.4 ± 0.1 | 0.4 ± 0.1 | 0.5 ± 0.1 | 0.7 ± 0.2 | 0.5 ± 0.1 | 0.5 ± 0.1 | *F* = 8.839  *P* < 0.05  η_p_^2^ = 0.639 | *F* = 2.319  *P* = 0.117  η_p_^2^ = 0.317 |  |
| ***Neutrophils*** |  |  |  |  |  |  |  |  |  |  | *F* = 0.549  *P* = 0.593 η_p_^2^ = 0.048 |
| Male | 3.8 ± 1.5 | 4.6 ± 2.6 | 4.1 ± 2.3 | 4.7 ± 2.4 | 3.7 ± 1.6 | 5.3 ± 2.9 | 4.0 ± 1.9 | 4.8 ± 2.0 | *F* = 7.807  *P* < 0.05  η_p_^2^ = 0.565 | *F* = 0.955  *P* = 0.403  η_p_^2^ = 0.137 |  |
| Female | 2.8 ± 0.7 | 3.9 ± 0.9 | 3.5 ± 0.8 | 4.4 ± 0.5 | 2.9 ± 0.8 | 4.7 ± 2.3 | 4.2 ± 2.2 | 5.5 ± 2.1 | *F* = 17.772  *P* < 0.01  η_p_^2^ = 0.780 | *F* = 1.013  *P* = 0.388  η_p_^2^ = 0.168 |  |
| ***Platelets*** |  |  |  |  |  |  |  |  |  |  |  |
| Male | 1.7 ± 0.5 | 1.9 ± 0.8 | 1.7 ± 0.6 | 1.7 ± 0.7 | 1.8 ± 0.6 | 2.1 ± 0.9 | 1.9 ± 0.7 | 1.8 ± 0.6 | *F* = 5.520  *P* < 0.05  η_p_^2^ = 0.479 | *F* = 0.509  *P* = 0.573  η_p_^2^ = 0.078 | *F* = 0.268  *P* = 0.742 η_p_^2^ = 0.02 |
| Female | 2.0 ± 0.2 | 2.3 ± 0.3 | 2.1 ± 0.3 | 2.0 ± 0.2 | 2.0 ± 0.2 | 2.6 ± 0.3 | 2.3 ± 0.4 | 2.0 ± 0.3 | *F* = 30.935  *P* < 0.01  η_p_^2^ = 0.861 | *F* = 2.779  *P* = 0.132  η_p_^2^ = 0.357 |  |

Values are mean ± SD. 0, 15, 60 refer to minutes post exercise. Of monocytes, < 10 % will be basophils and eosinophils. Leukocytes, lymphocytes, monocytes and neutrophils are presented as x10^9^/L and platelets are presented as x10^7^/L
